# Supplementary figures and images for: Autophagy-related genes prognosis signature as potential predictive markers for immunotherapy in hepatocellular carcinoma
Source: PeerJ. 2020 Jan 17;8:e8383. doi: 10.7717/peerj.8383 (PMC6970541; doi:10.7717/peerj.8383)

Seven ATGs are associated with clinicopathological features of HCC in the TCGA dataset

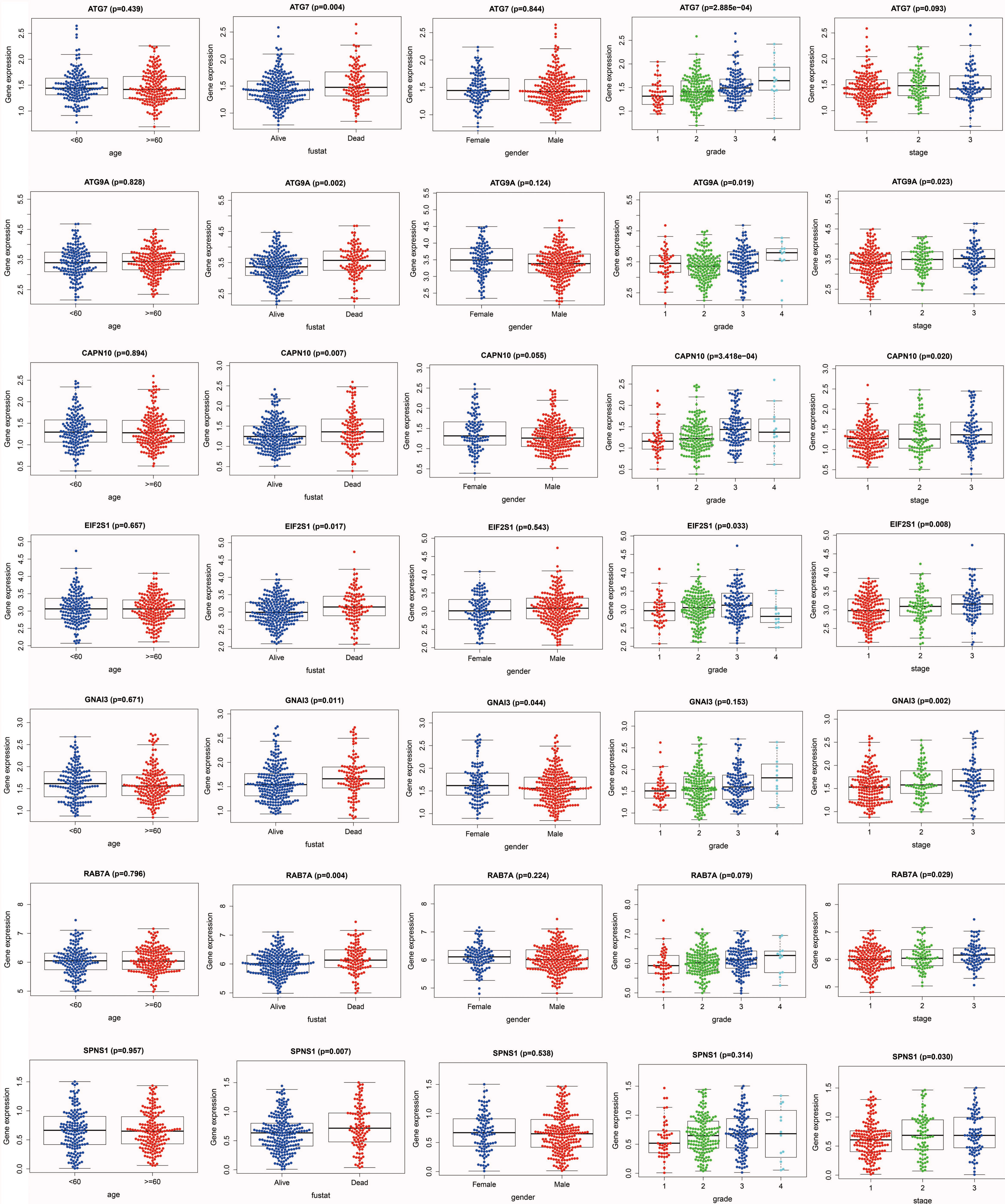

Supplement: Supplemental Information 1 [file peerj-08-8383-s001.pdf]

# Seven ATGs are associated with clinicopathological features of HCC in the ICGC dataset

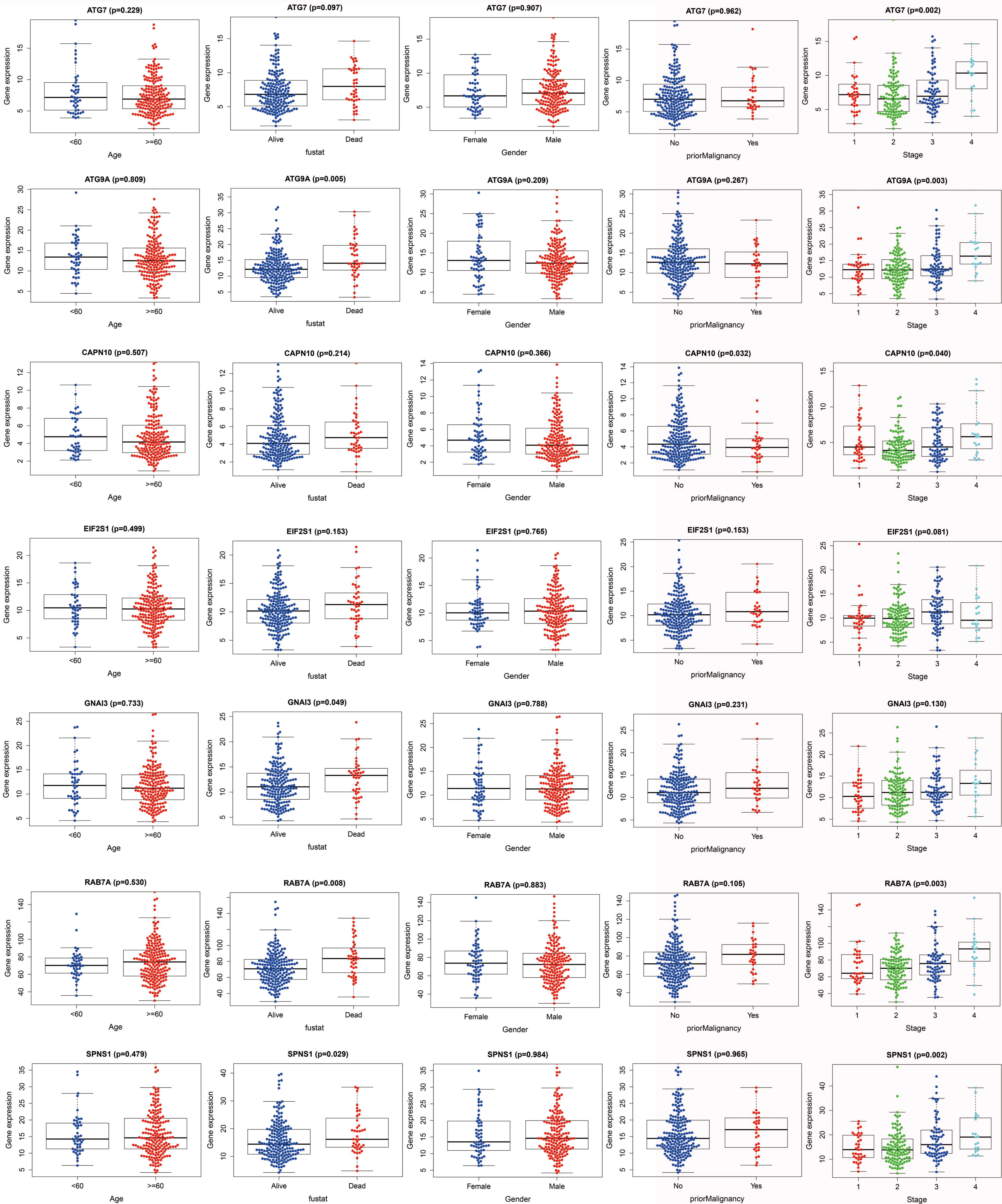

Supplement: Supplemental Information 2 [file peerj-08-8383-s002.pdf]

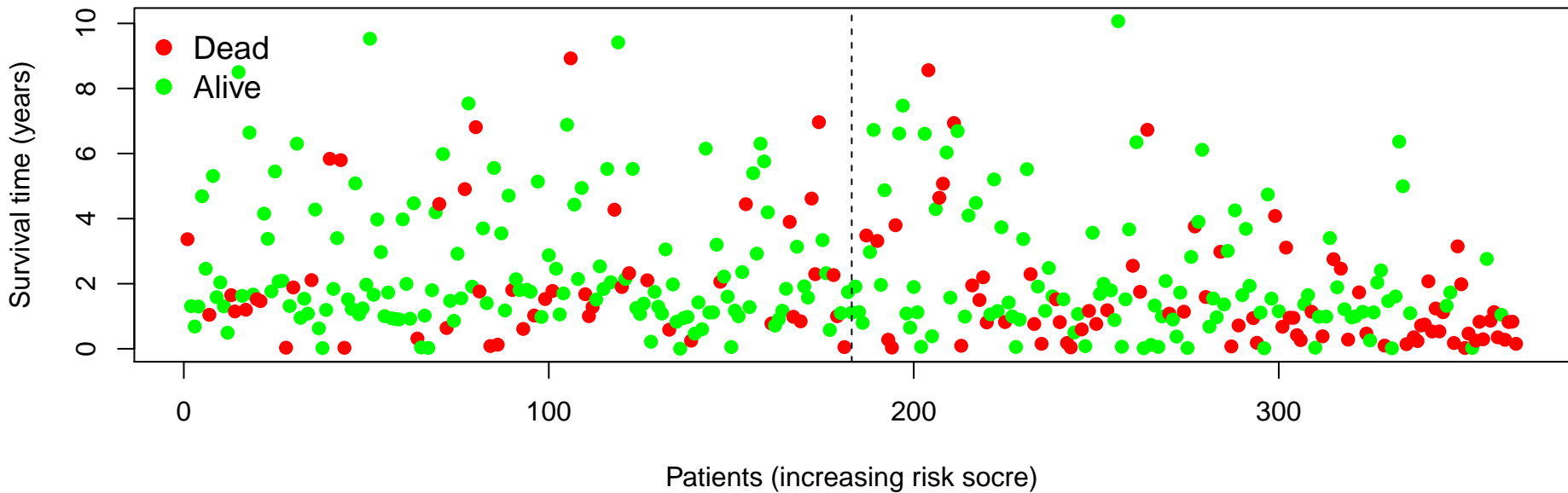

Supplement: Supplemental Information 3 [file peerj-08-8383-s003.pdf]

A

TCGA

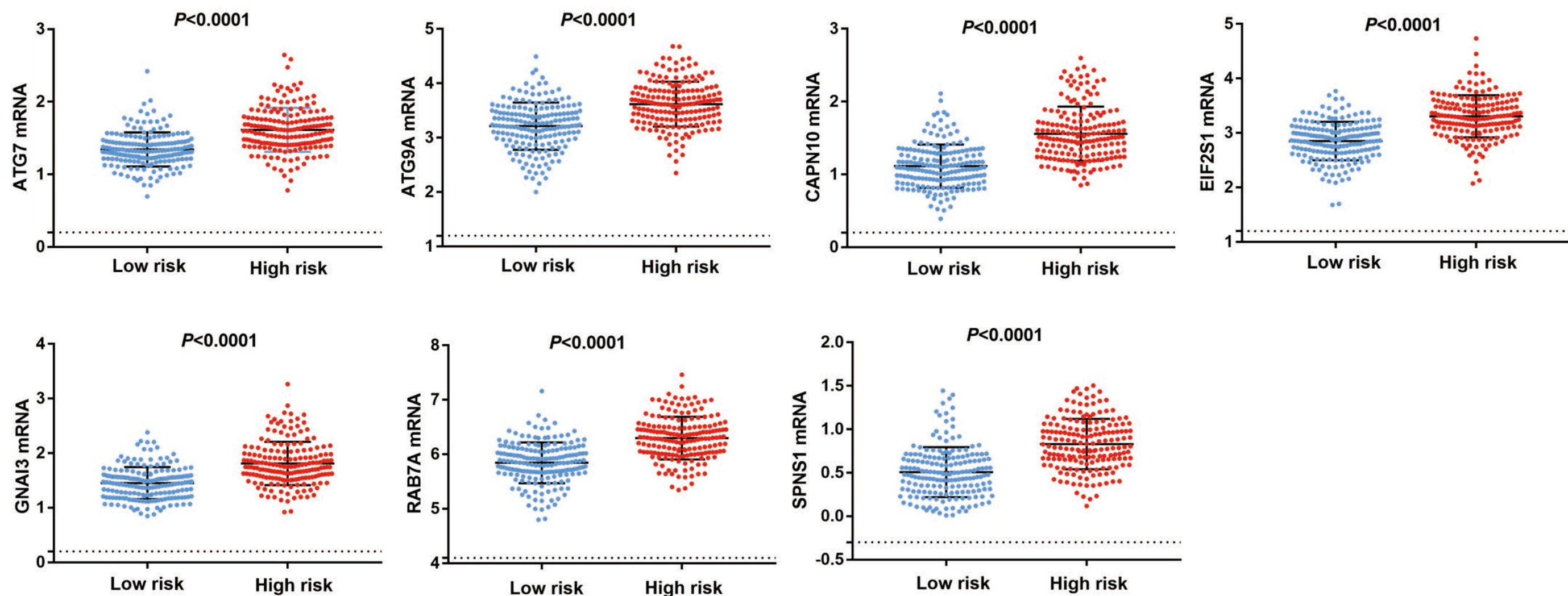

B

ICGC

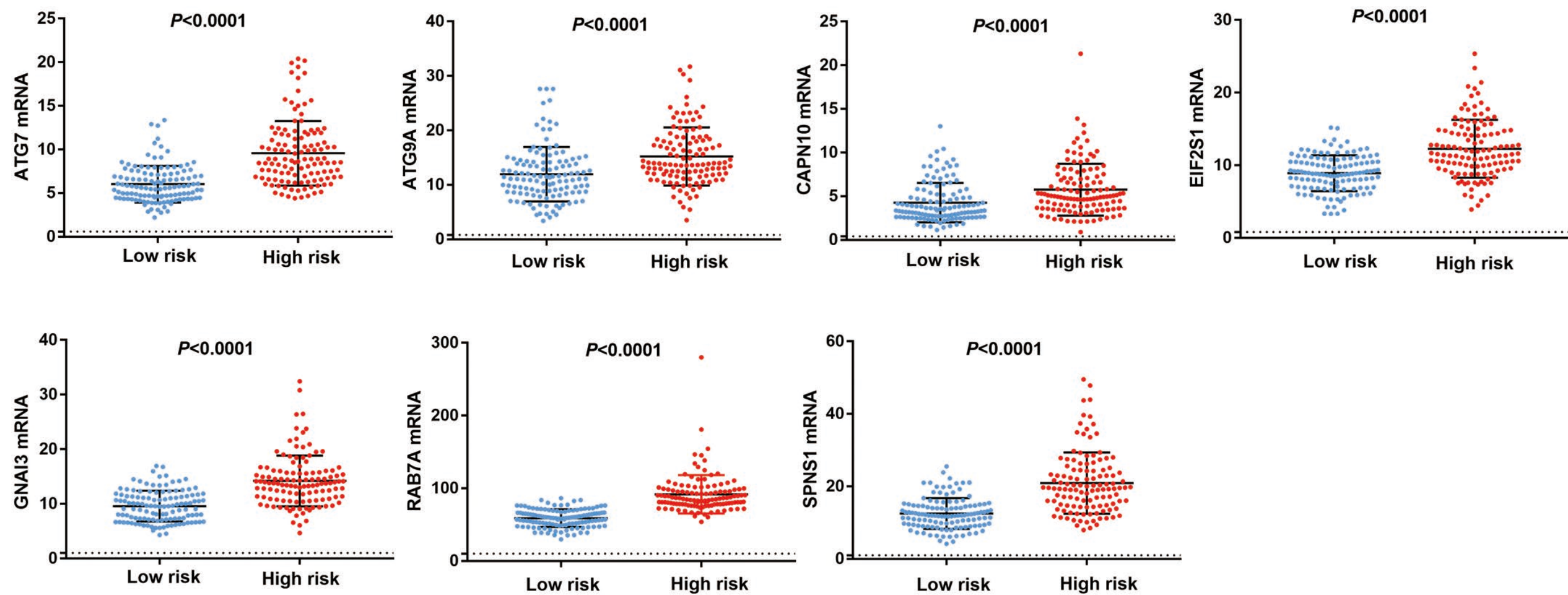

Supplement: Supplemental Information 4 [file peerj-08-8383-s004.pdf]

A

TCGA

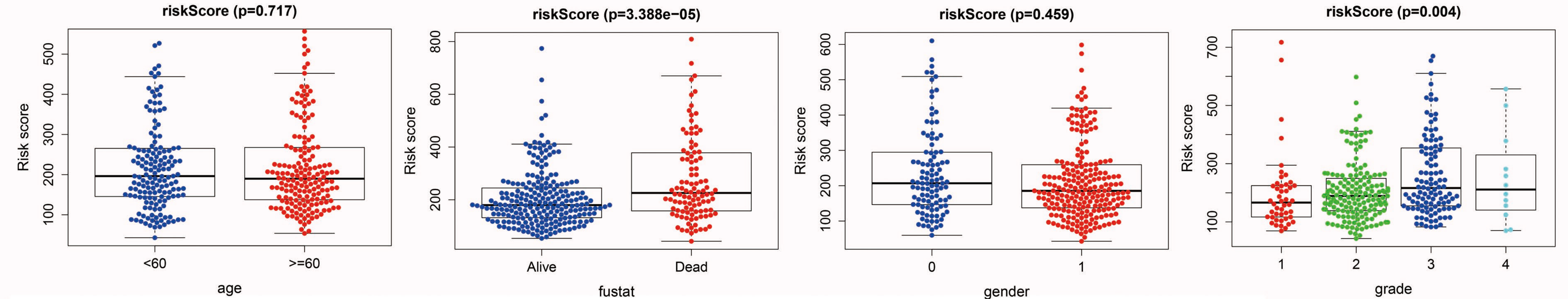

B

ICGC

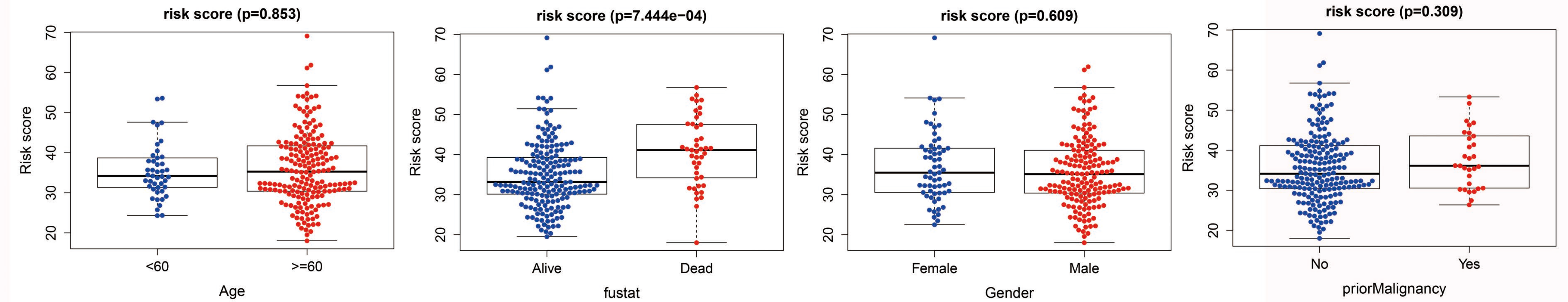

Supplement: Supplemental Information 5 [file peerj-08-8383-s005.pdf]

ICGC dataset

# CIBERSORT

Wilcoxon test  $P=0.012$

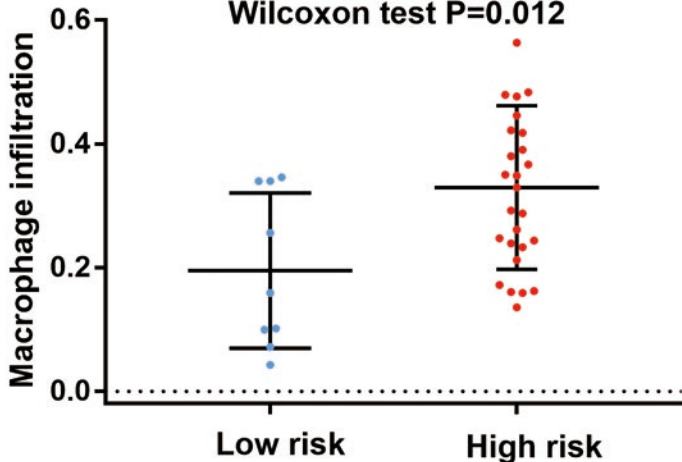

Supplement: Supplemental Information 7 [file peerj-08-8383-s007.pdf]
