# Supplementary material for: Autophagy-related genes prognosis signature as potential predictive markers for immunotherapy in hepatocellular carcinoma
Source: PeerJ. 2020 Jan 17;8:e8383. doi: 10.7717/peerj.8383 (PMC6970541; doi:10.7717/peerj.8383)
Supplement: Supplemental Information 6 [file peerj-08-8383-s006.pdf]

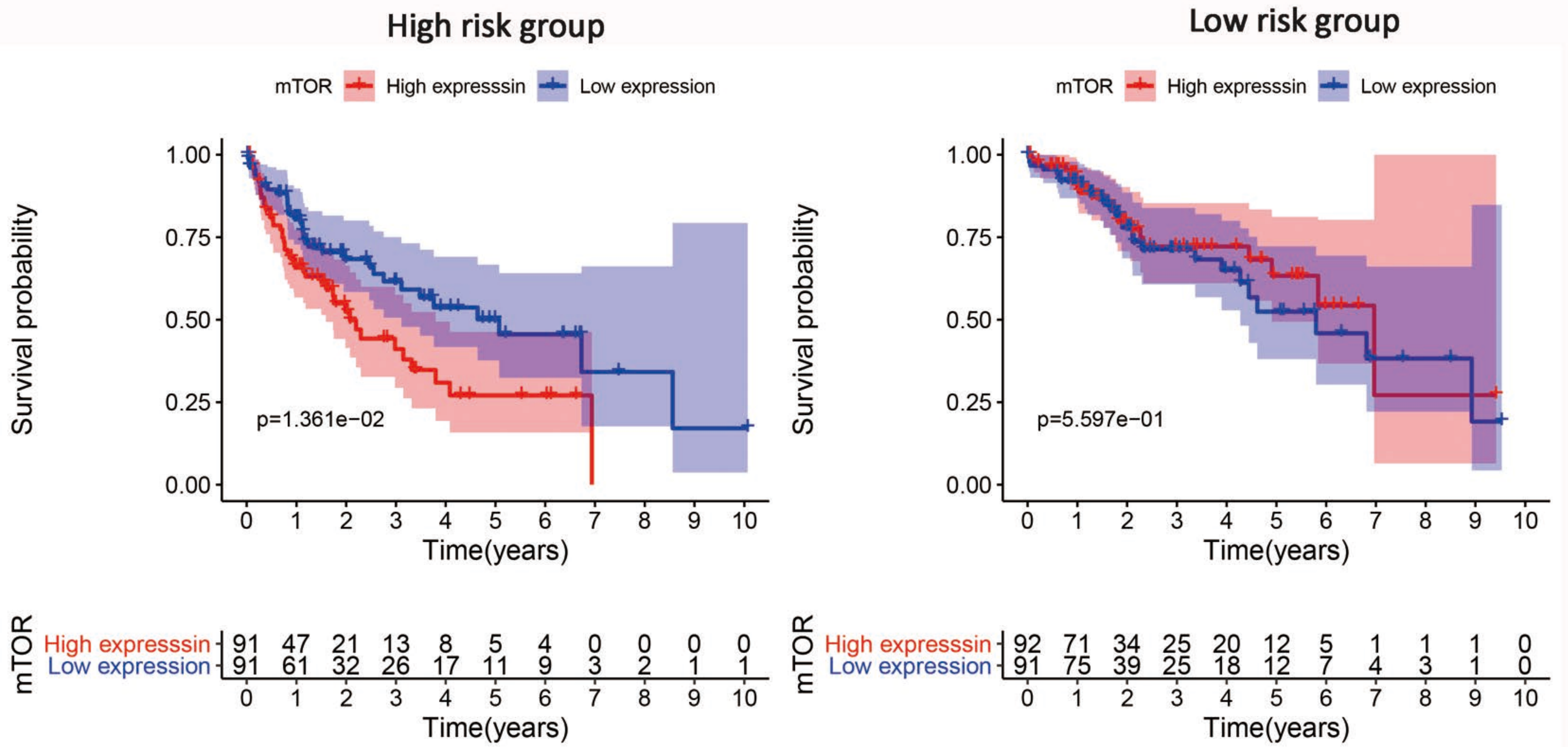
High risk group
mTOR
High expresssin
Low expression
Survival probability
1.00
0.75
0.50
0.25
0.00
p=1.361e−02
0 1 2 3 4 5 6 7 8 9 10
Time(years)
mTOR
High expresssin 91 47 21 13 8 5 4 0 0 0 0
Low expression 91 61 32 26 17 11 9 3 2 1 1
0 1 2 3 4 5 6 7 8 9 10
Time(years)
Low risk group
mTOR
High expresssin
Low expression
Survival probability
1.00
0.75
0.50
0.25
0.00
p=5.597e−01
0 1 2 3 4 5 6 7 8 9 10
Time(years)
mTOR
High expresssin 92 71 34 25 20 12 5 1 1 1 0
Low expression 91 75 39 25 18 12 7 4 3 1 0
0 1 2 3 4 5 6 7 8 9 10
Time(years)
